# Effectiveness and Safety of Bictegravir/Emtricitabine/Tenofovir Alafenamide in People Living with HIV Aged 50 Years and Older: A Retrospective Analysis of Naïve and Treatment-Experienced Individuals

**DOI:** 10.3390/v17111449

**Published:** 2025-10-31

**Authors:** Marcello Trizzino, Luca Pipitò, Floriana Di Figlia, Silvia Bonura, Federica Zimmerhofer, Andrea Cicero, Claudia Gioè, Antonio Cascio

**Affiliations:** 1 Infectious and Tropical Diseases Unit, Sicilian Regional Reference Center for the Fight Against AIDS, AOU Policlinico “P. Giaccone”, 90133 Palermo, Italy; marcello.trizzino@policlinico.pa.it (M.T.); luca.pipito@community.unipa.it (L.P.); floriana.difilglia@community.unipa.it (F.D.F.); silvia.bonura@policlinico.pa.it (S.B.); federica.zimmerhofer@community.unipa.it (F.Z.); andrea.cicero@community.unipa.it (A.C.); claudia.gioe@policlinico.pa.it (C.G.); 2 Palermo Fast-Track City, Casa dei Diritti, 90100 Palermo, Italy; 3 Department of Health Promotion, Mother and Child Care, Internal Medicine and Medical Specialties “G D’Alessandro”, University of Palermo, 90133 Palermo, Italy

**Keywords:** older people living with HIV, bictegravir, safety, immunological recovery, ≥50 years, metabolic safety, hepatic safety

## Abstract

**Background**: The aging of people living with HIV (PLWH) necessitates antiretroviral regimens (ART) with high efficacy, a favorable safety profile, and minimal drug–drug interactions. We evaluated the real-world performance of bictegravir/emtricitabine/tenofovir alafenamide (B/F/TAF) in PLWH aged ≥ 50 years, stratified by prior treatment experience. **Methods**: This retrospective cohort study included ART-naïve and virologically suppressed treatment-experienced PLWH aged ≥ 50 years who started B/F/TAF. Primary endpoints were virological suppression (HIV-1 RNA < 50 copies/mL) at 12 months ± 1 month and 24 months ± 1 month, and safety. Changes in immunological (CD4+ count, CD4+/CD8+ ratio), metabolic, and hepatic parameters were assessed. **Results**: Among 214 patients (37 naïve, 177 experienced; mean age 60.6 years), high virological suppression rates were observed in both naïve (85.7%) and experienced (93.9%) cohorts at 24 months. Immunologically, naïve patients experienced a robust increase in CD4+ counts (from 176 to 450 cells/μL, *p* < 0.001). A key finding was a significant increase in the CD4+/CD8+ ratio in the experienced cohort, which normalized from 0.95 at baseline to 1.12 at 24 months (*p* < 0.001). The regimen demonstrated a favorable safety profile: metabolic parameters remained stable, and hepatic enzymes significantly improved in naïve patients. Transient elastography confirmed no worsening of liver fibrosis or steatosis in experienced patients. The overall discontinuation rate was 19.2%, driven by different reasons between cohorts (e.g., comorbidities in naïve, strategic simplification in experienced). **Conclusions**: This real-world study confirms that B/F/TAF is a versatile cornerstone for the management of older PLWH. It demonstrates high efficacy in initiating treatment and is a safe, effective, and durable platform for treatment simplification. Its favorable metabolic and hepatic profile makes it particularly suitable for an aging population with a high burden of comorbidities, ensuring long-term treatment success.

## 1. Introduction

The remarkable success of modern antiretroviral therapy (ART) has transformed human immunodeficiency virus (HIV) infection from a fatal disease into a manageable chronic condition, leading to a significant increase in the life expectancy of people living with HIV (PLWH) [1,2].

Consequently, the global HIV population is aging, with the number of PLWH over 50 years old increasing at a faster rate than in the general population [2]. This demographic shift presents new clinical challenges, as older PLWH face an elevated burden of age-associated comorbidities, including cardiovascular, renal, and neurocognitive diseases, and cancer, often seen at an earlier age compared to their HIV-negative counterparts [1,3,4,5,6]. This heightened risk is linked to chronic inflammation and immune activation, which persist even with viral suppression [1,3,7].

The management of older PLWH is further complicated by the progressive accumulation of non-communicable diseases and the related challenge of polypharmacy [3,4,7]. This necessitates the use of antiretroviral regimens with a favorable safety profile and minimal drug–drug interactions (DDIs) to avoid adverse events and ensure long-term adherence and effectiveness. Bictegravir/emtricitabine/tenofovir alafenamide (B/F/TAF) is a single-tablet regimen known for its high efficacy, high barrier to resistance, and favorable tolerability. Recent studies have specifically explored the efficacy and safety of switching to B/F/TAF in virologically suppressed older patients, highlighting its potential to address the complexities of care in this population [8,9]. Despite this, clinical trial data, particularly in older individuals, remain limited, underscoring the need for further research in this area.

In this context, our study aims to evaluate the real-world effectiveness of a B/F/TAF-based regimen in a cohort of PLWH aged 50 or older, including both treatment-naive and experienced patients. We will specifically focus on immunological parameters, such as the CD4/CD8 ratio and lymphocyte typing, as well as metabolic aspects, multimorbidity, and polypharmacy, over 12 and 24 months.

## 2. Materials and Methods

### 2.1. Study Design and Population

This was a retrospective, observational, cohort study conducted using data from the Infectious Diseases Unit of Policlinico “Paolo Giaccone” database in Palermo. We screened all adult PLWH (≥18 years) who initiated a B/F/TAF-based regimen between 1 March 2019, and 31 March 2025. The study population was restricted to patients aged 50 years or older at baseline (B/F/TAF initiation). Patients were then categorized into two mutually exclusive cohorts:

1. Naïve Cohort: Patients who were ART-naïve and started B/F/TAF as their first-ever HIV treatment.
2. Experienced Cohort: Patients who were on a previous antiretroviral regimen for at least 3 months prior to switching to B/F/TAF. Virologic suppression at the time of switch was not required.

The main exclusion criterion for analysis was the absence of follow-up data. Notably, co-infection with Hepatitis B or the lack of a complete resistance history were not grounds for exclusion in this real-world cohort.

### 2.2. Study Objectives and Endpoints

The primary outcome was to evaluate the real-world virological effectiveness and safety of B/F/TAF in PLWH aged ≥ 50 years. HIV virological suppression was defined as having a HIV-1 RNA < 50 copies/mL and virological effectiveness was defined as the proportion of patients in both the naïve and experienced cohorts achieving HIV-1 RNA < 50 copies/mL at the primary timepoints of 12 months ± 1 month (M12) and 24 months ± 1 months (M24). HIV virological failure was defined as a persistent plasma HIV RNA level ≥ 200 copies/mL after at least 6 months of ART.

Secondary outcomes included:

- Immunological efficacy evaluated by change from baseline to M12 and M24 in CD4+ T-cell count and the CD4+/CD8+ ratio.
- Metabolic Safety evaluated by change from baseline to M12 and M24 in fasting glucose and the Triglyceride-Glucose (TyG) Index. The TyG index is a simple, low-cost biochemical marker of insulin resistance, calculated using the formula: Ln [fasting triglycerides (mg/dL) × fasting glucose (mg/dL)/2] [10].
- Hepatic Safety evaluated by change from baseline to M12 and M24 in liver enzymes (AST, ALT) and the Fibrosis-4 index (FIB-4) index. The FIB-4 is a non-invasive calculation using a patient’s age, AST, ALT, and platelet count to estimate liver fibrosis, or scarring [11]. Hepatic steatosis and fibrosis were also evaluated by change from baseline to M12 in liver stiffness (kPa) and Controlled Attenuation Parameter (CAP) score, assessed by transient elastography (FibroScan) in a subset of experienced patients.
- Treatment durability and tolerability profile. Long-term treatment durability, defined as the time from initiation to discontinuation of B/F/TAF, was evaluated using Kaplan–Meier survival analysis over the entire study period, and tolerability was concurrently assessed, with specific reasons for discontinuation described.

### 2.3. Data Collection and Variables

The following variables were collected from electronic medical records. At baseline, demographic information included age, sex, and ethnicity. HIV disease characteristics comprised the duration of HIV infection and antiretroviral treatment status, categorized as treatment-naïve or treatment-experienced; for the latter, details regarding prior therapeutic regimens were recorded. Data on co-infections included Hepatitis B surface antigen (HBsAg) and Hepatitis C virus (HCV) serostatus. Information on comorbidities and concomitant medications was also obtained, including the presence of multimorbidity, defined as the coexistence of three or more non-communicable diseases, and polypharmacy, defined as the concurrent use of five or more medications. Laboratory parameters included comprehensive virological (HIV-1 RNA), immunological (CD4+ T-cell count, CD4+/CD8+ ratio), metabolic (triglycerides), renal (creatinine), and hepatic (AST and ALT) panels. They were collected at baseline and during follow-up visits at months 12 and 24. Information regarding treatment discontinuation, including the date and reason for discontinuation as well as details of subsequent antiretroviral regimens, was collected. Any recorded adverse event was also documented.

### 2.4. Statistical Analysis

Categorical variables were described as frequencies and percentages. Continuous variables were summarized as mean ± standard deviation or median [interquartile range] based on distribution normality (assessed by the Shapiro–Wilk test). Baseline characteristics were compared between cohorts using Chi-square/Fisher’s exact tests for categorical variables and *t*-tests/Mann–Whitney U tests for continuous variables. Changes in continuous parameters from baseline were analyzed using paired *t*-tests or Wilcoxon signed-rank tests. Treatment durability was analyzed using Kaplan–Meier curves, and comparisons between cohorts were made using the log-rank test. A two-sided *p*-value < 0.05 was considered statistically significant. All analyses were performed using SPSS version 26.

### 2.5. Handling of Missing Data

For virological and immunological endpoints, patients with missing data at a given timepoint were excluded from the denominator for that analysis. This approach, known as an as-observed or on-treatment analysis, provides a conservative estimate of the regimen’s effectiveness among patients who remained in care and with available monitoring. No data imputation techniques were applied. This method was chosen to reflect real-world clinical practice, where follow-up can be variable. The number of patients with available data at each timepoint is explicitly reported for all analyses.

## 3. Results

### 3.1. Patient Population and Baseline Characteristics

A total of 214 PLWH initiated B/F/TAF during the study period. Baseline characteristics were available for all participants, whereas follow-up data were available for 173 individuals (80.8%), who were therefore included in the effectiveness analysis. The average age was 60.6 ± 7.3 years. Baseline characteristics of the cohort are summarized in Table 1. It was characterized by a high burden of age-related comorbidities and polypharmacy, reflecting the complexity of the contemporary aging HIV population. Of these, 37 (17.3%) were ART-naïve and 177 (82.7%) were treatment-experienced individuals who switched to B/F/TAF.

The overall population was predominantly male (74.8%) and of Caucasian ethnicity (91.6%). The prevalence of HCV and HBV co-infections was 23.4% and 6.5%, respectively, with no major differences between cohorts. The burden of non-communicable diseases was high, and multi-morbidity (≥3 conditions) was highly prevalent in both groups (56.3% in naïve vs. 63.3% in experienced). Dyslipidemia was more frequent in the experienced group (51.2% vs. 21.9%), as was osteoporosis (0% vs. 25.9%). The most common reason for switching in the experienced cohort was simplification/optimization of therapy (19.8%), followed by toxicity prevention (13.6%).

Previous antiretroviral regimens before switching to B/F/TAF included integrase inhibitor-based therapies in 101 patients (57.1%), of whom 58 (32.8%) were receiving older integrase inhibitors, namely raltegravir or elvitegravir. Protease inhibitor-based regimens were used in 37 patients (20.9%), and non-nucleoside reverse transcriptase inhibitor-based regimens in 21 patients (11.9%). A total of 18 patients (10.2%) were receiving combined regimens, including protease inhibitor plus integrase inhibitor in 14 cases (8.0%), integrase inhibitor plus non-nucleoside reverse transcriptase inhibitor in 3 cases (1.7%), and protease inhibitor plus maraviroc in 1 case (0.6%).

The median follow-up time on B/F/TAF was similar between the groups (Naïve: 3.28 years; Experienced: 3.44 years). The discontinuation rate was higher in the naïve cohort (27.0%) compared to the experienced cohort (17.5%).

### 3.2. Effectiveness Outcomes

Virological response rates are detailed in Table 2. As expected, no ART-naïve individuals were virologically suppressed at baseline, whereas 89.0% (129/145) of the treatment-experienced individuals were suppressed on their prior regimen before switching to B/F/TAF. Among naïve individuals, virological suppression was achieved in 80.8% (21/26) at 12 months. Of these, 85.7% (18/21) maintained suppression at 24 months. In the experienced cohort, virological suppression was 91.4% (117/128) at 12 months and increased to 93.9% (92/98) at 24 months.

### 3.3. Immunological Outcome

Immunologically, treatment with B/F/TAF was associated with an improvement in both ART-naïve and -experienced individuals. In the former, due to immune reconstitution, the average CD4+ count increased from 176.4 to 449.5 cells/μL at 24 months (*p* < 0.001), and the CD4+/CD8+ ratio improved markedly from 0.28 to 0.73 (*p* < 0.001).

In treatment-experienced individuals, CD4+ counts remained stable, and the CD4+/CD8+ ratio increased significantly from 0.95 at baseline to 1.12 at 24 months (*p* < 0.001), Table 3.

### 3.4. Metabolic and Hepatic Outcomes

Fasting glucose, TyG index, and hepatic markers are shown in Table 4. In ART-naïve individuals, fasting glucose and the TyG index (a marker of insulin resistance) remained stable over 24 months, whereas in treatment-experienced individuals, a transient increase in fasting glucose was observed at 12 months (*p* = 0.036), which returned to baseline levels by 24 months. In experienced individuals, a significant and sustained improvement in insulin sensitivity was observed, as evidenced by a progressive decline in the TyG index up to 24 months (*p* = 0.005).

Regarding hepatic safety, in naïve individuals, initiation of therapy led to significant reductions in AST and ALT levels at 12 months, with a concurrent significant improvement in the FIB-4 score at 24 months (*p* = 0.024). In experienced individuals, minor changes at 24 months were observed in AST (*p* < 0.001) and FIB-4 (*p* = 0.004), which were not indicative of hepatotoxicity.

Liver stiffness and CAP showed a long-term hepatic safety of B/F/TAF. As detailed in Table 5, baseline measurements showed no significant fibrosis and only mild steatosis in the cohort. After 12 months of treatment, no statistically significant changes in liver stiffness or CAP score were observed compared with baseline.

### 3.5. Treatment Durability, Safety, and Adverse Events

**Figure 1:**
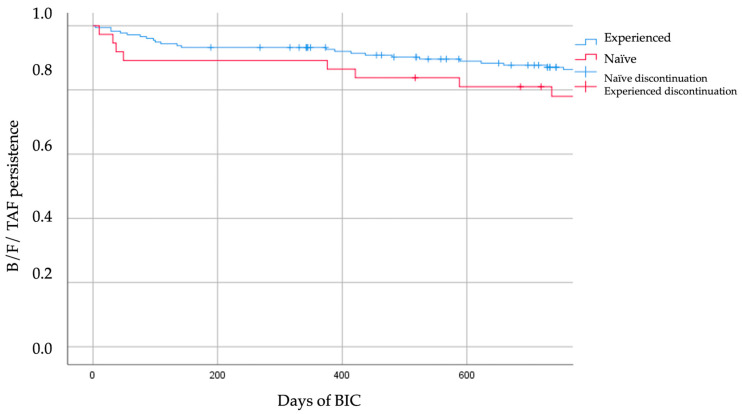
Kaplan–Meier curve for time to discontinuation of B/F/TAF at 24 months, stratified by prior treatment experience.

The long-term durability of B/F/TAF was assessed using Kaplan–Meier survival analysis, with treatment discontinuation as the event of interest (Figure 1). At 24 months, treatment persistence was 78.4% (29/37) in ART-naïve individuals and 87.6% (155/177) in treatment-experienced individuals. The log-rank test showed no statistically significant difference in discontinuation rates between the cohorts (*p* = 0.143). Over the 3.4-year study period, 73.0% (27/37) and 82.5% (146/177) of ART-naïve and treatment-experienced individuals, respectively, continued the B/F/TAF regimen. Causes of discontinuation and ART switches from B/F/TAF to a new regimen are reported in Table 6. Treatment with B/F/TAF was generally well-tolerated. Among the nine patients (22.0% of all discontinuations) who discontinued due to toxicity, the most frequently reported adverse events were nonspecific gastrointestinal disorders (n = 6), e.g., nausea, abdominal discomfort, followed by mild neuropsychiatric events (n = 3), including insomnia and dizziness. No discontinuations due to renal toxicity, significant bone mineral density changes, or other severe adverse events were reported.

Only two patients discontinued B/F/TAF due to virological failure. One patient in the treatment-naïve cohort subsequently continued antiretroviral therapy with darunavir/cobicistat plus dolutegravir, while the other, in the treatment-experienced cohort, switched to dolutegravir and doravirine. None of these patients developed resistance to bictegravir. However, the genotypic resistance test in the treatment-naïve individual revealed the H51Y mutation, which conferred intermediate resistance to cabotegravir, raltegravir, and elvitegravir. Thirteen patients discontinued B/F/TAF because they preferred to switch to long-acting injectable cabotegravir–rilpivirine therapy (CAB-RPV LA).

## 4. Discussion

This real-world study provides a comprehensive evaluation of the effectiveness, safety, and durability of B/F/TAF in a cohort of PLWH aged 50 years and older, encompassing both ART-naïve and treatment-experienced individuals. The principal findings demonstrate that B/F/TAF is a robust therapeutic option for this aging population, achieving high rates of virological suppression, promoting immunological benefits, and exhibiting a favorable metabolic and hepatic safety profile over a median follow-up of 3.4 years.

The high prevalence of multimorbidity (62.1%) and polypharmacy (29.0%) at baseline underscores that the participants represent a complex population with significant concomitant health challenges. B/F/TAF demonstrated its utility not in a selected clinical trial population but in a real-world setting, where DDIs and comorbidity management are daily concerns for clinicians.

Our results confirm the high antiviral efficacy of B/F/TAF, consistent with findings from randomized clinical trials and other real-world studies [8,9]. In naïve individuals, suppression rates of 80.8% and 85.7% at 12 and 24 months, respectively, are commendable in a real-world setting, reflecting the regimen’s high genetic barrier and ease of use. In the treatment-experienced cohort, maintenance of virological suppression in over 91% of patients reinforces B/F/TAF as a reliable option for treatment simplification, a strategy particularly relevant for older adults with polypharmacy [7].

A previous Italian study retrospectively evaluated a cohort of PLWH aged ≥ 60 years who switched to B/F/TAF. The most common reasons for switching were DDIs, especially with statins, antidepressants, cardiologic drugs, proton pump inhibitors, and benzodiazepines. After switching to B/F/TAF, the number of potential DDIs decreased by over 80%, while high virological suppression rates and good tolerability were maintained [12]. Wang et al., in a review, confirmed the low level of DDIs for modern integrase strand transfer inhibitors such as bictegravir, which primarily show significant interactions with polyvalent cations and strong CYP3A4/UGT1A1 inducers (e.g., rifampin, rifabutin, rifapentine, primidone, phenobarbital, St. John’s wort, carbamazepine, oxcarbazepine, and phenytoin) [13]. Furthermore, a pharmacokinetic study in ten male patients aged 50 years or older, without hepatic or renal impairment and with suppressed HIV RNA, who switched to B/F/TAF, demonstrated no correlations between age and any pharmacokinetic parameters [14]. These results were comparable to findings in younger HIV-negative participants from a previous study, indicating that B/F/TAF may be safely used in older patients. In addition, body weight, transaminases, renal function, lipid profiles, and bone mineral density remained stable over a 48-week follow-up period, except for one case of prerenal acute renal failure secondary to acute congestive heart failure [14].

Ekobena et al. observed a non-clinically significant increase in bictegravir exposure among older people living with HIV, which did not require clinical intervention [15]. Similar findings were obtained using physiologically based pharmacokinetic modeling in the Swiss HIV Cohort Study, demonstrating that dose modulation of B/F/TAF is not necessary in older PLWH [16].

In line with our findings, the BICOLDER study showed that B/F/TAF was a safe and effective switch strategy in a cohort of 24 PLWH aged 65 years or older. Mild adverse events were reported in six participants, with only one discontinuation. Moreover, this study also did not observe changes from baseline in weight, comorbidities, kidney parameters, cardiovascular risk, or frailty scores at 48 weeks [17]. A larger American cohort, including 350 individuals aged 50 years or older who switched to B/F/TAF, showed that 94% maintained HIV-1 RNA < 50 copies/mL, while 6% had levels between 50 and 400 copies/mL. Most previous DDIs were resolved, and treatment-related adverse events occurred in 15% of patients, with only eight discontinuations [18].

A large cohort of individuals aged over 50 years living with HIV, comprising 96 treatment-naïve and 450 treatment-experienced participants, was analyzed by Kityo et al. At Week 240, virological suppression was achieved in 98.5% of treatment-naïve and 93.6% of treatment-experienced participants. Treatment-related adverse events were reported in fewer than one-third of participants aged 50 years or older, with a low discontinuation rate of less than 5% [19].

A key and novel finding of our study is the significant immunological improvement observed, particularly in the treatment-experienced cohort. While CD4+ counts remained stable, the CD4+/CD8+ ratio increased significantly, normalizing from 0.95 at baseline to 1.12 at 24 months. This is of paramount importance, as an inverted CD4+/CD8+ ratio is a marker of persistent immune activation and immunosenescence, both associated with an increased risk of non-AIDS-defining comorbidities in older PLWH [1]. Our data suggest that switching to B/F/TAF may confer an immunological advantage beyond virological control, potentially contributing to healthier aging. This finding aligns with the growing focus on optimizing immunological health as a therapeutic goal in geriatric HIV care [7]. Gidari et al., in a cohort of naïve and experienced PLWH, including 86 ART-experienced individuals aged 60 years or older, reported both virological suppression and immunological improvement. They observed increases in CD4 cell count (from 528 to 581 cells/mm^3^) and the CD4+/CD8+ ratio (from 0.9 to 1.0) under B/F/TAF [20]. A mild increase (+0.02) in the CD4+/CD8+ ratio was also reported in PLWH switching from dual therapy to B/F/TAF with detectable viral load (HIV-RNA ≥ 50 copies/mL) [21].

The aging HIV population faces a high burden of metabolic diseases, making the safety profile of ART critically important [3]. Our study demonstrates a reassuring metabolic safety profile for B/F/TAF. The stability of fasting glucose and the TyG index in naïve patients, together with a significant improvement in insulin sensitivity (TyG index) in the experienced cohort, addresses concerns regarding potential metabolic complications. This is a strong argument in favor of B/F/TAF, especially compared with older PI-based regimens [22].

Furthermore, the hepatic safety data are compelling. The significant reductions in liver enzymes and FIB-4 score in naïve patients likely reflect the resolution of HIV-related hepatic inflammation and steatosis following effective ART initiation. Importantly, the objective assessment via transient elastography in experienced patients provided direct evidence of the regimen’s long-term hepatic safety, showing no progression of liver fibrosis or steatosis. This is crucial for an aging population with a high prevalence of nonalcoholic fatty liver disease and other chronic liver conditions. Our previous work reported a significant reduction in liver steatosis, as assessed by FibroScan, in a cohort of 25 individuals on BIC/F/TAF [23].

The overall discontinuation rate of B/F/TAF was 19.2% over a median follow-up of 3.4 years. Only 9 individuals discontinued for toxicity, and of these, none developed a severe adverse event related to the therapy.

No discontinuations were attributed to renal adverse events in either cohort. These findings suggest that the regimen is well-tolerated in patients with aging-related comorbidities and polypharmacy, where renal safety is often a critical consideration. The absence of clinically significant kidney-related adverse events supports the suitability of B/F/TAF for long-term use in a diverse population of PLWH. Furthermore, recent findings support the use of once-daily B/F/TAF in PLWH and in patients with end-stage kidney disease on hemodialysis [24].

A notable difference emerged when stratifying by prior treatment experience: the discontinuation rate was higher in the ART-naïve cohort (27.0%) compared with the treatment-experienced cohort (17.5%), as detailed in Table 6. This disparity highlights distinct clinical scenarios. In naïve patients, discontinuations occurred earlier and were primarily driven by factors such as death and DDIs, reflecting the complex clinical profile of patients presenting late for care. In contrast, discontinuations in the experienced cohort were more often related to long-term tolerability or, notably, strategic treatment optimization. A significant finding was that 22.6% of discontinuations in the experienced group were for “simplification,” with the most frequent subsequent regimens being CAB-RPV LA (32.3% of new regimens) and DTG/3TC (29.0%). This pattern strongly suggests that B/F/TAF often serves as a highly effective and well-tolerated bridge to more advanced therapeutic options, rather than being discontinued due to failure. This observation is supported by recent evidence from the SOLAR trial, which demonstrated that switching from B/F/TAF to CAB-RPV LA was associated with significantly improved patient-reported outcomes, including treatment satisfaction and relief from psychological burdens related to HIV therapy [25,26]. Furthermore, CAB-RPV LA was a treatment option in individuals who faced persistent viremia, adherence difficulties, malabsorption syndromes, or psychosocial barriers [27].

The low incidence of virological failure (3.2% in experienced patients) with B/F/TAF further confirms the regimen’s high efficacy, while the discontinuation analysis provides a realistic depiction of its role within a dynamic treatment landscape for an aging population. Finally, no resistance to bictegravir was detected in patients who experienced virological failure. The patient in the treatment-naïve cohort, despite harboring the H51Y mutation conferring intermediate resistance to cabotegravir, raltegravir, and elvitegravir, did not develop resistance to bictegravir. These findings underscore that B/F/TAF remains a robust treatment option with a high genetic barrier, even in the rare cases of virological failure.

### Limitations

Our study has several limitations. Its retrospective and single-center design may limit generalizability. The sample size, particularly for the naïve cohort, reduces the statistical power for some subgroup analyses. The lack of a control group prevents causal inferences, and the reasons for “Patient Choice” discontinuations could not be further delineated. Furthermore, for 41 individuals described at baseline, no follow-up information was available, and they were therefore excluded from the analysis. Despite these limitations, the study provides valuable real-world insights into the use of B/F/TAF in a well-characterized cohort of older PLWH.

## 5. Conclusions

In conclusion, this study demonstrates that, in a real-world cohort of older PLWH with multimorbidity and polypharmacy, B/F/TAF is a highly effective, safe, and durable regimen. Its ability to deliver these outcomes in a complex clinical environment supports its role as a key therapeutic option for promoting healthier aging in PLWH. Its ability to promote immunological normalization, coupled with its neutral metabolic and favorable hepatic profile, makes it a particularly suitable choice for addressing the complex needs of the aging HIV population.

Importantly, our real-world data position B/F/TAF as a pivotal agent in the modern treatment sequence. For many patients, it serves as an excellent platform for transitioning to even more innovative options, such as long-acting injectable regimens, when this step aligns with patient preferences and clinical goals. Therefore, B/F/TAF represents a valuable tool in the continuous effort to ensure not only longer, but also healthier and more patient-centric lives for older people living with HIV.

## Figures and Tables

**Table 1:** Baseline Characteristics of the Study Population, Stratified by Prior Treatment Experience.

Characteristic	Overall Population (n = 214)	Naïve Cohort (n = 37)	Experienced Cohort (n = 177)
Demographics			
Age, years, mean ± SD	60.6 ± 7.3	58.6 ± 6.7	61.1 ± 7.4
Male Sex, n (%)	160 (74.8%)	30 (81.1%)	130 (73.4%)
Ethnicity, n (%)			
Caucasian	196 (91.6%)	33 (89.2%)	163 (92.1%)
African	18 (8.4%)	4 (10.8%)	14 (7.9%)
HIV-Related Factors			
HIV History, years, mean ± SD	*	N/A	20.9 ± 9.6
HCV Seropositivity, n/N (%)	50/214 (23.4%)	6/37 (16.2%)	44/177 (24.9%)
HBsAg Seropositivity, n/N (%)	14/214 (6.5%)	3/37 (8.1%)	11/177 (6.2%)
Comorbidities, n/N (%)			
Dyslipidemia	93/200 (46.5%)	7/32 (21.9%)	86/168 (51.2%)
Hypertension	86/210 (41.0%)	11/35 (31.4%)	75/175 (42.9%)
Osteoporosis	43/198 (21.7%)	0/32 (0.0%)	43/166 (25.9%)
Obesity	27/199 (13.6%)	6/33 (18.2%)	21/166 (12.7%)
Diabetes Mellitus	20/208 (9.6%)	2/34 (5.9%)	18/174 (10.3%)
Oncological Disease	27/199 (13.6%)	2/32 (6.3%)	25/167 (15.0%)
COPD	10/199 (5.0%)	0/32 (0.0%)	10/167 (6.0%)
Current Smoking	95/209 (45.5%)	15/36 (41.7%)	80/173 (46.2%)
Multi-morbidity (≥3 NCDs)	123/198 (62.1%)	18/32 (56.3%)	105/166 (63.3%)
Polypharmacy (≥5 meds)	36/124 (29.0%)	5/13 (38.5%)	31/111 (27.9%)
Treatment Data			
Years on B/F/TAF, median [IQR]	3.43 [2.37–5.38] (n = 173)	3.28 [2.69–4.58] (n = 27)	3.44 [2.22–5.46] (n = 146)
Discontinued B/F/TAF, n (%)	41 (19.2%)	10 (27.0%)	31 (17.5%)

N refers to patients who did not discontinue treatment. NCDs: Non-Communicable Diseases. N/A: Not Applicable (statistical testing pending). * The mean value was not calculated for the entire population, as it would be influenced by the inclusion of treatment-naïve PLWH, for whom the time variable is considered as 0 years. IQR interquartile range.

**Table 2:** Virological response to B/F/TAF in naïve and experienced individuals over time.

Timepoint	Naïve Cohort (n = 27)	Experienced Cohort (n = 146)
Baseline (Pre-switch)		
HIV-RNA < 50 copies/mL, n/N (%)	0/27 (0%)	129/145 (89.0%)
12 Months		
HIV-RNA < 50 copies/mL, n/N (%)	21/26 (80.8%)	117/128 (91.4%)
No Virological Suppression (HIV-RNA ≥ 50), n	5	11
No Virological Data, n	1	18
24 Months		
HIV-RNA < 50 copies/mL, n/N (%)	18/21 (85.7%)	92/98 (93.9%)
No Virological Suppression (HIV-RNA ≥ 50), n	3	6
No Virological Data, n	6	48

**Table 3:** Immunological response to B/F/TAF in naïve and experienced patients. Analysis includes only patients who did not discontinue B/F/TAF. Denominators (N) for virological response represent patients with available data at each timepoint. *p*-values from the Wilcoxon signed-rank test for paired comparisons vs. baseline. A significant *p*-value (*p* < 0.05) indicates a statistically significant change from baseline; for the CD4+/CD8+ ratio, this is observed in both cohorts and reflects a significant increase.

Parameter	Timepoint	Naïve Cohort (n = 27)	*p*-Value	Experienced Cohort (n = 146)	*p*-Value
Immunological Response					
CD4+ Count (cells/μL), mean ± SD	Baseline	176.4 ± 180.9	-	662.7 ± 389.5	-
	12 Months	402.6 ± 273.9	<0.001	641.4 ± 351.2	0.391
	24 Months	449.5 ± 270.7	<0.001	680.4 ± 390.9	0.225
CD4+/CD8+ Ratio, mean ± SD	Baseline	0.28 ± 0.28	-	0.95 ± 0.76	-
	12 Months	0.57 ± 0.43	<0.001	1.02 ± 0.86	<0.001
	24 Months	0.73 ± 0.52	<0.001	1.12 ± 1.02	<0.001

**Table 4:** Metabolic and hepatic safety parameters in treatment-naïve and experienced individuals on B/F/TAF. Data are presented as mean ± standard deviation. *p*-values from the Wilcoxon signed-rank test for paired comparisons vs. baseline. A significant *p*-value (*p* < 0.05) indicates a statistically significant change from baseline.

Parameter	Timepoint	Naïve Cohort (n = 27)	*p*-Value	Experienced Cohort (n = 146)	*p*-Value
Metabolic Parameters					
Fasting Glucose (mg/dL), mean ± SD	Baseline	98.5 ± 28.7	-	96.6 ± 28.4	-
	12 Months	93.2 ± 11.9	0.983	109.1 ± 94.3	0.036
	24 Months	90.4 ± 14.3	0.690	98.8 ± 26.5	0.061
TyG Index, mean ± SD	Baseline	4.72 ± 0.30	-	4.69 ± 0.25	-
	12 Months	4.67 ± 0.37	0.352	4.67 ± 0.29	0.061
	24 Months	4.67 ± 0.25	0.084	4.64 ± 0.25	0.005
Hepatic Parameters					
AST (U/L), mean ± SD	Baseline	35.9 ± 30.1	-	22.4 ± 10.9	-
	12 Months	24.2 ± 10.7	0.020	23.0 ± 7.9	0.068
	24 Months	23.6 ± 6.9	0.101	25.4 ± 11.5	<0.001
ALT (U/L), mean ± SD	Baseline	33.7 ± 35.3	-	23.3 ± 12.8	-
	12 Months	20.9 ± 12.2	0.028	25.1 ± 12.5	0.191
	24 Months	22.9 ± 11.7	0.484	25.9 ± 14.6	0.248
Liver Fibrosis Index					
FIB-4 score, mean ± SD	Baseline	2.90 ± 4.80	-	1.40 ± 0.67	-
	12 Months	1.57 ± 0.68	0.183	1.39 ± 0.60	0.153
	24 Months	1.48 ± 0.90	0.024	1.51 ± 0.92	0.004

**Table 5:** Liver stiffness and steatosis assessment by transient elastography in treatment-experienced individuals. Data are presented for the subset of patients with available FibroScan measurements at both timepoints. *p*-values from the Wilcoxon signed-rank test for paired comparisons vs. baseline.

Parameter	Baseline (n = 105)	12 Months (n = 36)	*p*-Value
Liver Stiffness (kPa), mean ± SD	5.99 ± 2.99	6.88 ± 3.79	0.191
CAP Score (dB/m), mean ± SD	246.42 ± 66.45	249.24 ± 61.41	0.383

CAP: Controlled Attenuation Parameter.

**Table 6:** Discontinuations of B/F/TAF, reasons for stopping, and subsequent regimens.

Parameter	Overall (N = 41)	Naïve Cohort (n = 10)	Experienced Cohort (n = 31)
Total Discontinuations, n (% of cohort)	41 (19.2%)	10 (27.0%)	31 (17.5%)
Reason for Discontinuation, n (% of column discontinuations)			
Patient Choice	13 (31.7%)	3 (30.0%)	10 (32.3%)
Toxicity	9 (22.0%)	1 (10.0%)	8 (25.8%)
Simplification	7 (17.1%)	0 (0.0%)	7 (22.6%)
Failure	2 (4.9%)	1 (10.0%)	1 (3.2%)
DDI (Drug–Drug Interaction)	2 (4.9%)	2 (20.0%)	0 (0.0%)
CV Prevention	5 (12.2%)	1 (10.0%)	4 (12.9%)
Death	2 (4.9%)	2 (20.0%)	0 (0.0%)
Median Time to Discontinuation, days	-	352.5	437.0
New Antiretroviral Regimen Prescribed, n			
CAB-RPV (Long-Acting)	13	3	10
DTG/3TC	9	0	9
DTG + DOR	6	1	5
DOR + TAF/FTC	2	0	2
DRV/b + RAL	2	0	2
DOR/TDF/3TC	2	1	1
DTG + TAF/FTC	1	0	1
DRV/b + DTG	1	1	0
DRV/c/TAF/FTC	1	0	1
DTG + TDF/FTC	1	1	0
EFV/TDF/FTC	1	1	0
None (Interruption)	2	2	0

‘Simplification’ refers to switching to a simpler regimen (e.g., 2-drug or long-acting). DDI: Drug–Drug Interaction; CV: Cardiovascular. Regimens are listed in descending order of frequency in the overall cohort. CAB cabotegravir; c cobicistat; DOR doravirine; DRV darunavir; DTG dolutegravir; EFV efavirenz; FTC emtricitabine; RAL raltegravir; RPV rilpivirine; TAF tenofovir alafenamide; TDF tenofovir disoproxil fumarate; 3TC lamivudine.

## Data Availability

Data are available on request to the corresponding author.

## Abbreviations

The following abbreviations are used in this manuscript:

3TC	lamivudine
ART	antiretroviral therapy
B/F/TAF	bictegravir/emtricitabine/tenofovir alafenamide
CAB-RPV LA	Cabotegravir–rilpivirine therapy
CAP	Controlled Attenuation Parameter
DDIs	drug–drug interactions
DTG	dolutegravir
FIB-4	Fibrosis-4 index
HBsAg	Hepatitis B surface antigen
HCV	Hepatitis C virus
HIV	human immunodeficiency virus
PLWH	People living with HIV
TyG	Triglyceride-Glucose index
